# Mild Amnestic Cognitive Impairment and Depressive Symptoms in Autoimmune Encephalitis Associated with Serum Anti-Neurexin-3α Autoantibodies

**DOI:** 10.3390/brainsci11060673

**Published:** 2021-05-21

**Authors:** Niels Hansen, Claudia Lange, Fabian Maass, Lina Hassoun, Caroline Bouter, Winfried Stöcker, Björn Hendrik Schott, Jens Wiltfang, Dirk Fitzner

**Affiliations:** 1 Department of Psychiatry and Psychotherapy, University Medical Center Göttingen, Von-Siebold-Str. 5, 37075 Goettingen, Germany; claudia.lange@gwdg.de (C.L.); lina.hassoun@med.uni-goettingen.de (L.H.); bjoernhendrik.schott@med.uni-goettingen.de (B.H.S.); jens.wiltfang@med.uni-goettingen.de (J.W.); 2 Department of Neurology, University Medical Center Göttingen, Robert-Koch Str. 40, 37075 Goettingen, Germany; fabian.maass@med.uni-goettingen.de; 3 Department of Nuclear Medicine, University Medical Center Göttingen, Robert-Koch Str. 40, 37075 Goettingen, Germany; caroline.bouter@med.uni-goettingen.de; 4 Euroimmun Reference Laboratory, Seekamp 31, 23650 Luebeck, Germany; w.stoecker@euroimmun.de; 5 Neurosciences and Signaling Group, Institute of Biomedicine (iBiMED), Department of Medical Sciences, University of Aveiro, 3820-293 Aveiro, Portugal; 6 German Center for Neurodegenerative Diseases (DZNE), Von-Siebold-Str. 3a, 37075 Goettingen, Germany

**Keywords:** neurexin-3α antibody, mild cognitive impairment, autoimmune encephalitis, depression

## Abstract

(1) Background: autoimmune encephalitis associated with neurexin-3α antibodies is a seldom reported disease entity often accompanied by a severe clinical neuropsychiatric syndrome. (2) Method: we report on the case of a 58-year-old man diagnosed with neurexin-3α-associated autoimmune encephalitis revealing cognitive decline and depression before the proof of neurexin-3α antibodies. He underwent neuropsychological testing, peripheral blood and cerebrospinal fluid analysis, neuroimaging and electroencephalography. (3) Results: our patient’s main clinical feature was amnestic cognitive decline in combination with depressive symptoms. CSF analysis showed elevated phosphorylated tau protein 181 and positive proof of serum neurexin-3α antibodies in a cell-based assay. An 18F-FDG-PET/CT of the brain initially showed bilateral cerebral hypometabolism prefrontal and parietal, which was absent in follow up. The brain MRI was unremarkable. EEG recordings showed frontotemporal slowing in the theta and delta range. (4) Conclusions: taken together, we assumed autoimmune encephalitis associated with serum neurexin-3α antibodies. To the best of our knowledge, we are the first to report on a predominantly mild clinical manifestation entailing amnestic mild cognitive impairment in addition to depression, thus broadening the clinical spectrum associated with neurexin-3α antibodies.

## 1. Introduction

Autoimmune encephalitis is a rapidly evolving disease condition often associated with a variety of membrane surface and intracellular antibodies. Diverse neural and intracellular autoantibodies were recently reported to be associated with cognitive impairment [1,2]. A novel type of autoimmune encephalitis was previously described as associated with an autoantibody against the extracellularly localized neurexin-3α, clinically characterized by a preceding infectious-like prodrome accompanied by headaches and gastrointestinal symptoms, followed by a rather rapid development of seizures, memory impairment, confusion, loss of consciousness, central hypoventilation, abnormal behavior, and speech disturbances [3,4]. Neurexin-3α is a trans-synaptic membrane protein with an intra- and extra-cellular site involved in many synaptic brain functions and synapse formation. Furthermore, it is involved in regulating neurotransmitter release at presynaptic sites via calcium coupling to exocytosis [5,6], indicating its impact on neural transmission and its relevance to cognitive functioning reliant on synaptic interactions. Animal studies suggest [3,5] that autoantibodies against the neurexin-3α antigen may lead to cognitive dysfunction. However, cognitive dysfunction was not reported until now as the first and predominant symptom in neurexin-3α disease. Here we present the case of a patient with autoimmune encephalitis associated with neurexin-3α antibodies and prominent amnestic mild cognitive impairment as the leading symptomatology. We thereby aim to enlarge the phenotypic spectrum of cognitive disorders associated with neurexin-3α antibodies to inspire memory clinics to perform adequate testing and to offer such patients early immunotherapy, as there is solid evidence that immunotherapy (i.e., rituximab) can lead to a complete remission of symptoms in neurexin-3α disease [7].

## 2. Case Report

### 2.1. Clinical Presentation

**Figure 1:**
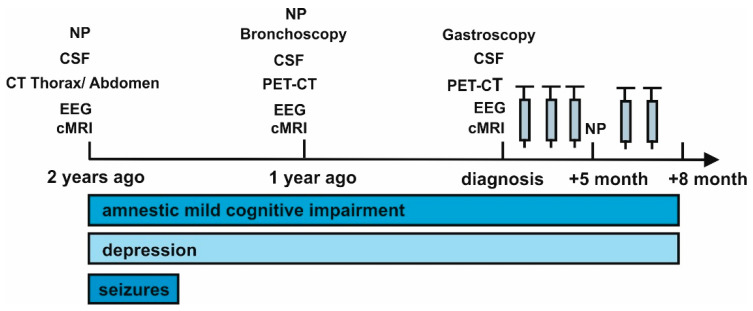
Time course of symptoms evolution. Abbreviations: CSF = cerebrospinal fluid, cMRI = cranial magnetic resonance imaging, CT = computed tomography, EEG = electroencephalography, NP = neuropsychological testing, PET CT = positron emission tomography computed tomography, electroencephalography, and PET CT = positron emission tomography computed tomography. The gray syringes indicate the corresponding methylprednisolone cycles.

We report on a 58-year-old man who presented with memory disturbances for two years and word-finding difficulties of a progressive nature (Figure 1).

He also suffered a depressive syndrome that began developing dynamically two years ago. Two years ago, he suffered several complex focal and secondary generalized seizures that were triggered by a gastrointestinal infection involving elevated leucocytes and calcitonin gene-related peptide (Table 1).

Later, temporal lobe epilepsy was diagnosed and was likely the cause of the reported seizures. No new seizures were reported to date. Two years ago, he developed psychomotor retardation, loss of drive, depressive mood, and personality changes revealing behavioral abnormalities that persisted as variably expressed symptoms diagnosed as major depressive disorder.

### 2.2. Patient History and Examination

In addition to the aforementioned symptoms, his latest neurological examination revealed apathy, slightly elevated muscle tone without atrophy, and dysmetria in finger-noise trials on both sides, as well as pallhypaesthesia in both legs. The psychiatric examination revealed a depressive syndrome with mood dysfunction, libido loss, loss of drive, and disordered vital sensations.

His family anamnesis revealed autoimmune disorders, namely, of his six siblings: one sister suffered from rheumatoid arthritis and another from multiple sclerosis. His father died of unknown causes at 60 years of age. He has no children and is married. In addition to his cognitive impairment, he suffers from restless-legs syndrome, sleep apnea, coronary heart disease, steatosis hepatis, hyperlipoproteinemia, and nicotine dependence.

Antidepressive medication was started two years ago with citalopram (20 mg/d) and quetiapine (75 mg/d), followed by a later withdrawal of these drugs due to their inefficacy. He was prescribed duloxetine one year later and continued with it, leading to some alleviation of his depressive symptoms.

### 2.3. Neuropsychological, Electroencephalographic, Neuroimaging and Biosamples Laboratory Data

He began undergoing thorough neuropsychological testing two years ago (Table 1). The first neuropsychological investigation revealed deficits in the learning and consolidation of verbal material, as well as deficits in letter and semantic word fluency. Assuming normal speech lateralization, we diagnosed a left-sided frontotemporal dysfunction two years ago. We observed a small improvement in his verbal memory capacity and semantic and phonemic word fluency a year ago in a follow up neuropsychological investigation. However, his latest examination at follow up 1, five months after diagnosis, showed a mild progression concurring with his neuropsychological status two years ago. His MMSE total score was 27 and we diagnosed an amnestic mild cognitive impairment (aMCI).

Furthermore, his latest MRI was unremarkable, although an MRI scan taken a year ago showed prominent signal changes in the left temporal lobe, showing a reason for his temporal lobe epilepsy. Blood measurements revealed elevated levels of neuron-specific enolase one year ago, which remained elevated in a control measurement. CYFRA was elevated one year ago in the blood, but normalized at diagnosis (Table 1).

Cerebrospinal fluid (CSF) analysis showed elevated phosphorylated tau protein with 70 pg/mL (pathological: >61 pg/mL, Table 1). In comparison to one year ago, both CSF tau and phosphorylated tau protein decreased (Table 1).

Furthermore, autoantibody analysis in serum and CSF showed positive serum and CSF paraneoplastic Yo antibodies and positive serum SOX1 antibodies in immunoblots. Both Yo and SOX1 antibodies revealed borderline positivity in immunoblots, whereas neurexin-3α antibodies showed a seropositivity in a cell-based assay (1:100). In addition to a cell-based assay, an immunofluorescent test was used to detect anti-neurexin-3α antibodies. Anti-Yo and SOX1 antibodies were sought in BIOCHIP mosaics with brain tissue and recombinant cells. However, we observed no anti-Yo, SOX1 or neurexin-3α reactivity in the tissue immunoreactivity analysis.

His electroencephalography (EEG) showed, repeatedly over two years, intermittent slowing in the theta to delta range located in the bi-frontotemporal region.

### 2.4. 18F-FDG-PET Investigation

Our detection of paraneoplastic antibodies and Yo seropositivity a year ago prompted his latest whole body positron emission tomography computed tomography (PET-CT) to look for cancer. The initial whole body 18F-FDG-PET/CT showed increased uptake in the axillary lymph nodes. Lymph node dissection in the left axilla was performed and no malignancy was detected histopathologically. Furthermore, a bronchoscopy was performed and revealed no signs of an endotracheal or endothoracic tumor. A whole body 18F-FDG-PET/CT was repeated one year later and showed increased uptake in the distal esophagus, which was histologically identified as Barrett’s esophagus without any sign of malignancy.

**Figure 2:**
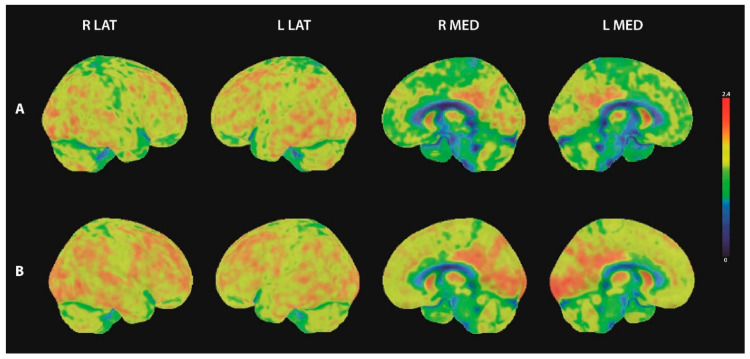
18F-FDG-PET images of the brain. Three-dimensional surface projections of FDG-uptake in lateral and medial view. (**A**) initial 18F-FDG-PET scan showed bilateral hypometabolism prefrontal medial and parietal, (**B**) follow up 18F-FDG-PET showed physiological cerebral FDG uptake. R lat = right lateral view; L lat = left lateral view; R med = right medial view; L med = left medial view.

The initial 18F-FDG-PET/CT of the brain showed distinct bilateral hypometabolism prefrontal and parietal (Figure 2A). In addition, statistical image analysis based on the 3D-SSP method (using Cortex ID Suite, GE Healthcare) showed pathological results within the precuneus region. A follow up brain 18F-FDG-PET/CT one year after the initial scan showed physiological cerebral FDG uptake with marginal pathological results in the statistical image analysis within the right prefrontal and right parietal inferior regions (Figure 2B).

### 2.5. Diagnosis of Autoimmune Encephalitis and Therapeutic Treatment

The clinical features of cognitive impairment, in addition to a depressive syndrome and word-finding difficulty, in conjunction with detected serum autoantibodies (anti-neurexin-3α, anti-Yo, and anti-SOX1), intermittent frontotemporal slowing in EEG, borderline PET-CCT abnormalities in the precuneus, parietal and frontal region, and CSF antibodies (anti-Yo antibody) led us to the diagnosis of possible autoimmune encephalitis according to the Graus criteria [8] (Table 1). Serum neurexin-3α antibodies did not suggest a probable CNS autoimmune process as CSF neurexin-3α antibodies do. However, the clinical features entailing a subacute onset of depressive symptoms, mild cognitive impairment and seizures in addition to the diagnostic evidence (such as brain abnormalities in PET, and EEG functional disturbances in frontotemporal regions in conjunction with serum neurexin-3α antibodies) may be caused by underlying autoimmune encephalitis. Furthermore, neurexin-3α antibodies are membrane surface autoantibodies believed to be pathogenic that are also demonstrated in neuronal cell cultures [3].

Taken together, the evidence above suggests a possible role for neurexin-3α antibodies in our patient’s disease phenotype, although we could exclude that neurexin-3α plays an epiphenomenon in the context of the clinical phenotype. However, we acknowledge the limitation that neurexin-3α antibodies were unfortunately unconfirmed via positive seroreactivity on rodent brain tissues and are not detected in CSF. Arguments against a relevant role for anti-Yo and anti-SOX1 antibodies, in relation to our clinical phenotype, are the borderline reactivity and, in addition, the missing seroreactivity in brain tissues. Furthermore, antibodies against intracellular antigens are believed to non-pathogenic.

After diagnosis (Figure 1), high dosage intravenous methylprednisolone (1 g) was administered in five cycles over a three day period six weeks apart. The patient has thus far failed to demonstrate improvement in his cognitive dysfunction and depressive symptoms after immunotherapy at follow up 1 and follow up 2, at 5 months and 8 months after diagnosis, respectively (Figure 1).

## 3. Discussion

Our findings imply that the clinical phenotype of neurexin-3α disease can be expanded to include an initial aMCI as the leading symptom. 

### 3.1. Potential Role of Human Neurexin-3α for Memory and Cognition

An in vitro study demonstrated, via neuronal cell culture, that human neurexin-3α antibodies might interfere with the growth and formation of hippocampal synapses [3], indicating potential defective synaptic information processing and, likely, memory encoding within the hippocampus relevant for declarative memory formation. Animal models suggest that the extracellular epitope neurexin-3α seems to play a major role in the morphology of presynapses, and in the probability of neurotransmitters released at excitatory synapses in the hippocampus, thus affecting mainly presynaptic long-term plasticity [9]. However, other in vitro studies showed that neurexin-3α, with its extracellular site, regulates excitatory receptors, such as the α-amino-3-hydroxy-5-methyl-4-isoxazolpropionic acid receptor (AMPAR), at the postsynaptic site [10]. The postsynapse is crucial for the induction, maintenance, and reversal of long-term synaptic plasticity phenomena, such as long-term potentiation and long-term depression, as synaptic mechanisms of learning and memory. The aforementioned animal experiments suggest that neurexin-3α autoantibodies might impair signaling at glutamatergic synapses, resulting in defective memory formation in humans. In line with our hypothesis, animal studies showed that the formation of fear memory is associated with a significant repression of specific exons in the neurexin-3α gene [11], pointing towards neurexin-3α’s prominent role in generating a memory trace. An underlying mechanism for memory control by neurexin-3α might be the inhibition of AMPAR-mediated synaptic transmission via alternative splicing [12]. AMPAR are key regulators for modulating long-term synaptic plasticity and memory formation [13,14]. Concurring with these assumptions, a recent overview underlined the importance of neurexins to various cognitive functions and circuits [15], not just affecting memory functions.

### 3.2. Characterization of Brain Metabolism in Mild Cognitive Impairment Associated with Serum Anti-Neurexin-3α Antibodies

We observed many additional features in our patient that are typical for neurexin-3α disease, such as the absence of lesions in the brain MRI. However, the initial 18F-FDG-PET/CT showed distinct prefrontal and parietal hypometabolism. To the best of our knowledge, there are no studies on 18F-FDG-PET in autoimmune encephalitis associated with neurexin-3α autoantibodies. However, some studies have evaluated FDG-patterns in autoimmune encephalitis associated with other autoantibody types. While hypermetabolism in the medial temporal lobe has been described as characteristic for limbic encephalitis with LGI1 antibodies, patterns of selective hypometabolism in other cortical areas have been reported by several working groups [16,17,18,19,20]. Regional cerebral hypometabolism was detected in different brain regions (including frontal and parietal regions as in our patient), while patterns also differed between the autoantibody types involved [21]. The 18F-FDG-PET proved to be more sensitive than an MRI [18,19,20]. Therefore, an 18F-FDG-PET of the brain should be considered a valuable tool in the diagnostic workup of patients with suspected autoimmune encephalitis in addition to whole body imaging. Our patient’s follow-up 18F-FDG-PET revealed normal brain metabolism. Brain metabolism might change over the disease course, influenced potentially by the autoimmune response or other factors. To assess 18F-FDG-PET’s prognostic value during autoimmune encephalitis, more studies applying 18F-FDG-PET/CT with a longitudinal approach are needed.

It is unclear whether seizures appearing at the onset of our patient’s clinical manifestation are associated with neurexin-3α disease, as his temporal-lobe seizures demonstrated no temporal relationship with proven neurexin-3α antibodies. However, the interleukin-2 receptor in the serum might be an indicator of autoimmune activity [22] and, as it was elevated two years ago and coincided with his symptoms then, we cannot rule out an autoimmune process starting two years ago. We cannot exclude the possibility that the presence of serum SOX1-antibodies and Yo-antibodies modulated our patient’s disease course.

### 3.3. Axonal Neurodegeneration in Possible Autoimmune Encephalitis Associated with Neurexin 3α-Antibodies

Another interesting aspect was the elevated phosphorylated tau protein found in patients with early AD. Neurexin-3α is a protein found to be affected early in preclinical AD, as a recent investigation by Leo revealed [23], making an inflammation-triggered brain-degeneration process conceivable in our patient. However, our patient’s decrease in the tau protein level over time suggests reversible brain damage due to a presumed autoimmune process. Unlike cases reported by Gresa-Arribas et al. [3], our patient is only mildly affected, thus there is potential to prevent further brain damage through immunotherapy.

## 4. Conclusions

In conclusion, the search for neural autoantibodies is warranted in patients presenting with a combination of aMCI and depressive disorder, or in patients suffering mild aMCI alone. Our patient’s depression might explain the aMCI, as depressive disorders often are accompanied by cognitive dysfunction [24]. Therefore, it is more likely that our patient’s late-onset depression and aMCI are interlinked and might represent the same underlying disease entity associated with the serum neurexin-3α antibodies. This point of view is further corroborated by the fact that his depression has not been sufficiently responsive to standard antidepressive drugs, thus implying an organic origin (i.e., autoimmune) of depression. However, it cannot be fully excluded that the patient’s aMCI is due to a depressive disorder of no organic origin.

In summary, the neurexin-3α disease spectrum is likely to encompass aMCI and depressive mood within its first clinical manifestation.

## Figures and Tables

**Table 1:** Neuropsychological and laboratory data.

Laboratory Parameter	Laboratory Data				
	2 Years Ago	1 Year Ago	Diagnosis	Follow Up 5 Months	Follow Up 8 Months
**PB**					
CRP mg/L (<0.2)	12.2	3.2	8.3	-	6.0
Leukocytes 10^3^/μL (4–11)	14.4	9.25	8.59	-	9.8
CK (30–200 U/L)	248	-	-	-	-
***Immunologic marker***					
Rheumafactor (<15.9 IU/mL)	<10	-	-	-	-
Circulating immune complexes (<45 IU/mL)	<25	-	-	-	-
Complement C3c (0.82–1.93 g/L)	1.35	-	-	-	-
Complement C4 (0.15–0.57 g/L)	0.37	-	-	-	-
ANA/ENA (<0.7)	0.1	-	-	-	-
ACE (20–70 IU/L)	28	-	-	-	-
p-ANCA IF (<1:10)	negative	-	-	-	-
c-ANCA IF (<1:10)	negative	-	-	-	-
sIL-2R (223–710 IU/mL)	1378	-	-	-	-
ASMA (<1:100)	negative	-	-	-	-
ANA IF (<1:100)	1:320	-	-	-	-
***Tumor marker***					
ß-HCG IU/L (<0.9 IU/L)	-	-	0.9	-	-
CA 15-3 kU/L (<12.9 U/L)	-	-	31.3	-	-
CEA μg/L (<5 μg/L)	-	3.7	5	-	-
NSE μg/L (<18.3 μg/L)	-	26.3	18.3	-	-
S-100 μg/L (<0.15 μg/L)	-	-	0.15	-	-
PSA μg/L (<4 μg/L)	-	-	2.47	-	-
CYFRA 21-1 μg/L (<2.1 μg/L)	-	3.8	1.0	-	-
***Antibody***					
Yo	negative	+	+	-	-
SOX1	negative	negative	+	-	-
Neurexin-3alpha	-	-	+(1:100)	-	-
**CSF**					
***Antibody***					
Yo	negative	negative	negative	-	-
SOX1	negative	negative	negative	-	-
Neurexin-3alpha	-	-	negative	-	-
***Cells***					
Cells/μL (˂5 μg/L)	1	2	0	-	-
Lymphocytes %	66	-	91	-	-
Monocytes %	28	-	9	-	-
***Proteins***					
Albumin mg/L	507	338	591	-	-
IgG mg/L	54.8	45.4	65.1	-	-
IgA mg/L	6.3	4.5	7.8	-	-
IgM mg/L	1.2	1.1	1.5	-	-
QAlb %	14.8	8	14.1	-	-
QIgG %	6.2	4.4	7.3	-	-
QIgA %	4.3	2.3	4.1	-	-
QIgM %	1.6	1.2	1.8	-	-
***Specific antibody***					
Rubella-AI (<1.5 AI)	-	-	1.0	-	-
VZV-AI (<1.5 AI)	-	negative	1.1	-	-
HSV-AI (<1.5 AI)	-	0.9	1.1	-	-
EBV-AI (<1.5 AI)	-	0.7	0.8	-	-
CMV-AI (<1.5 AI)	-	negative	0.8	-	-
***Cell destruction marker***					
Tau protein pg/mL (<450 pg/mL)	-	536	353	-	-
P-Tau 181 protein pg/mL (<61 pg/mL)	-	90	70	-	-
ß-Amyloid 1–42 pg/mL (>450 pg/mL)	-	1728	1534	-	-
ß-Amyloid 1–40 pg/mL	-	17,743	18,777	-	-
ß-Amyloid 1–42/1–40 × 10 (>0.5)	-	0.97	0.82	-	-
***Neuropsychology***					
WAIS-IV Block Design	−0.7	-	-	-	-
WAIS-IV Matrix Reasoning	-	−0.7	-	-	-
TMT A	−1.0	0.0	-	−0.5	-
TMT B	0.4	0.7	-	0.1	-
WAIS-IV Coding	−0.7	0.0	-	−0.7	-
VLMT Immediate Recall	−2.0	−1.1	-	−2.0	-
VLMT Long Delay Free Recall	−1.0	−1.3	-	−1.6	-
WMS-IV Logical Memory I	−2.8	−2.1	-	−2.8	-
WMS-IV Logical Memory II	−2.8	−2.1	-	−2.8	-
WMS-IV Visual Reproduction I	−0.7	0.7	-	0.0	-
WMS-IV Visual Reproduction II	-	0.3	-	−1.0	-
WAIS-IV Digit Span forward	0.7	0.0	-	0.0	-
WAIS-IV Digit Span backward	0.3	0.3	-	−1.0	-
RWT Letter fluency	−1.5	1.0	-	−1.0	-
RWT Semantic fluency	−1.4	−1.9	-	−1.9	-
NAB Maze Test	-	1.9	-	-	-

Abbreviations: Alb = albumin, ACE = angiotensin-converting enzyme, ANA = antinuclear antibody, c-ANCA = cytoplasmatic-anti-neutrophil cytoplasmic antibodies, ASMA = anti-smooth muscle antibody, CEA = carcinoembryonic antigen, CMV = Cytomegalovirus, CRP = calcitonin gene related peptide, CSF = cerebrospinal fluid, CT = computer tomography, CYFRA = cytokeratin 19 fragment, EBV = Ebstein Barr Virus, EEG = electroencephalography, ENA = extractable nuclear antigen, HSV = Herpes Simplex Virus, IF = immunofluorescence, IgA = immunoglobulin A, IgG = immunoglobulin G, IgM = immunoglobulin M, Interleukin 2 R (sIL-2R) MMSE = Mini Mental Status Examination, MRI = magnetic resonance imaging, NAB = Neuropsychological Assessment Battery, NSE = neuron specific enolase, p-ANCA = perinuclear-anti-neutrophil cytoplasmic antibodies, PB = peripheral blood, PET-CCT = positron emissions tomography cranial computer tomography, PSA = prostate-specific antigen, P-Tau 181 = phosphorylated tau protein 181, Q = quotient, sIL-2R = soluble interleukin 2 receptor, RWT = Regensburger Verbal Fluency Test, TMTA/B = Trail Making Test A/B, ß-HCG = beta human chorionic gonadotropin, VLMT = Verbaler Lern- und Merkfähigkeitstest (German version of the Auditory Verbal Learning Test), VZV = Varizella Zoster Virus, WAIS-IV = Wechsler Adult Intelligence Scale- fourth edition, and WMS-IV = Wechsler Memory Scale-fourth edition. The values in the brackets after the items indicate the cut-off levels for pathological levels.

## Data Availability

Data are available from the corresponding authors.
